# The efficacy of vitamin B6 as an adjunctive therapy to lithium in improving the symptoms of acute mania in patients with bipolar disorder, type 1; a double‐blind, randomized, placebo‐controlled, clinical trial

**DOI:** 10.1002/brb3.2394

**Published:** 2021-10-17

**Authors:** Rahim Badrfam, Seyed‐Ali Mostafavi, Ali Khaleghi, Shahin Akhondzadeh, Atefeh Zandifar, Malihe Farid, Nami Mohammadian Khonsari, Mohammad Reza Mohammadi

**Affiliations:** ^1^ Psychiatry and Psychology Research Center Roozbeh Hospital Tehran University of Medical Sciences Tehran Iran; ^2^ Department of Psychiatry Roozbeh Hospital Tehran University of Medical Sciences Tehran Iran; ^3^ Social Determinants of Health Research Center Alborz University of Medical Sciences Karaj Iran; ^4^ Department of Psychiatry Imam Hossein Hospital Alborz University of Medical Sciences Karaj Iran; ^5^ Non communicable Diseases Research Center Alborz University of Medical Sciences Karaj Iran; ^6^ Student Research Committee Alborz University of Medical Sciences Karaj Iran

**Keywords:** anthropology, bipolar disorder, homocysteine, lipids, mania, sleep, vitamin B6

## Abstract

**Objective:**

Vitamin B6 has been linked to a variety of probable roles, including anti‐inflammatory, homocysteine‐lowering, serotonin‐regulating, and dopamine‐lowering. In this study, we investigated the possible effect of vitamin B6 on bipolar disorder in manic episode with psychotic feature in a placebo‐controlled double‐blind clinical trial in a psychiatric hospital.

**Methods:**

This study was performed on 50 patients who were equally divided into two groups (each group included 25 patients) using 80 mg of vitamin B6 daily or placebo. At the beginning and end of the study, they were evaluated for lab tests, inflammatory biomarkers and level of blood homocysteine. Also, at the baseline and in weeks 2, 4, and 8, they were evaluated based on the anthropometric measurements, score obtained from the Young Mania Questionnaire, Mini‐Mental State Examination (MMSE), and the Pittsburgh Sleep Questionnaire.

**Results:**

Accordingly, based on Yang Mania scoring scale, no significant difference was observed between the two groups receiving vitamin B6 and placebo (22.68 ± 5.39 vs. 21.80 ± 5.39 [*p*‐value = .51]). Based on MMSE, significant improvement in cognitive status was obtained in group placebo compared to vitamin B6 group (25.24 ± 1.96 vs. 24.40 ± 3.25, respectively [*p*‐value = .01]). At the Pittsburg scale (total, there was no statistically significant difference between the two groups receiving vitamin B6 and placebo (1.04 ± 0.20 vs. 0.48 ± 0.50 [*p*‐value = .23]). Additionally, no significant difference was observed between the two groups regarding the anthropometric status.

**Conclusions:**

According to this study, the daily dose of 80 mg of vitamin B6 for 8 weeks in patients with bipolar disorder in the manic episode with psychotic feature treated daily with lithium, was not associated with a significant improvement in mood status compared to the control–placebo group. It is recommended to perform similar studies in a multi‐center manner with a larger sample size and longer duration.

## Abstract 2

The daily dose of 80 mg of vitamin B6 for 8 weeks in patients with bipolar disorder in the manic episode with psychotic feature treated daily with lithium, was not associated with a significant improvement in mood status compared to the control–placebo group.

## 1 INTRODUCTION

Bipolar disorder type I is one of the most debilitating psychiatric disorders (Phillips & Vieta, 2007). In this severe mood disorder, manic or depressed episode leads to disability of the individual and social functioning of the patient (Rosa et al., 2010). Aggression, disinhibited behaviors and disturbed sleep, are some of the symptoms of manic episodes of this disorder (Smedler et al., 2020). Despite some differences in the results of some studies, the lifetime prevalence of this disorder estimates 3.3% and its 12‐month prevalence as equal to 2% (Sadocket al., 2017).In some studies, the prevalence rate of unipolar mania among patients with bipolar disorder has been reported to be 5% to 7.2% (Baek et al., 2014). According to some estimates, 0.4%of total disability‐adjusted life years (DALYs) and 1.3% of the total years lived with disability (YLDs) can be attributed to bipolar disorder (Ferrari et al., 2016).

Some patients with type 1 bipolar disorder experience symptoms such as distraction and cognitive impairments such as decreased concentration and attention (Konstantakopoulos et al., 2016; Sanchez‐Autet et al., 2018).Also, a group of patients with this disorder suffer from the psychotic feature of this disease. The severity of symptoms in the acute phase of the disease in such a condition is high, although there is disagreement about its long‐term prognosis compared to disorders without psychotic feature (Burton et al., 2018).

The treatment for bipolar disorder is mainly the use of mood stabilizing medication (Fountoulakis et al., 2005). One of the most important side effects of treatment with some mood stabilizing medication such as lithium is overweight and metabolic and endocrine effects (including lipid profile disorders) (Belcastro et al., 2013; Livingstone & Rampes, 2006).It seems that the use of compounds that, in addition to being useful in controlling the symptoms of mania, reduce the side effects of these drugs, is very beneficial (Zheng et al., 2018).

In bipolar disorder, especially during manic episodes, the level of gamma amino butyric acid decreases and the level of glutamate rises; these can cause restlessness and irritability as well as sleep disorders in these patients (Daniele et al., 2012; Moradi et al., 2018).According to a number of studies, some nutritional supplements and vitamins can play an auxiliary and effective role in the treatment of mental disorders. Studies have examined the effects of factors such as vitamin B12, vitamin C, folate, lecithin, and omega‐3s on these disorders (Lakhan & Vieira, 2008; Mitchell et al., 2014; Patrick & Ames, 2015; Qureshi & Al‐Bedah, 2013). Also, vitamin B6 is one of the main cofactors in biochemical reactions that regulate cellular metabolism and is effective in many physiological processes (Parra et al., 2018). Vitamin B6 is a water‐soluble vitamin that is resistant to heat and acid; the coenzyme state of this vitamin is pyridoxal phosphate (PLP), which is obtained from pyroxidine, pyridoxal, and pyridoxamine (various forms of vitamin B6) in the presence of phosphate with the help of pyridoxal kinase and has a very important role in biochemical reactions, including protein (amino acids) metabolism and neurotransmitters (gamma‐aminobutyric acid, serotonin, etc.) and regulation of glycogen phosphorylase, etc. (Zhao, 2011).

Vitamin B6 in conditions such as restlessness and irritability, by increasing the production of GABA and other mechanisms, can prevent the occurrence or exacerbation of the above symptoms (Huang et al., 2016). Homocysteine levels rise in manic episode in bipolar disorder (Chiarani et al., 2013). Vitamins B6, B12, and folic acid reduce homocysteine levels in people with bipolar disorder. Vitamin B6 may also improve cognitive symptoms (Malouf & Evans, 2003; Selhub, 2002). In bipolar disorder, we see an increase in dopamine levels in the manic episode (Spinneker et al., 2007; Tang & Wei, 2004). Also, the regulation of serotonin is impaired (Qureshi & Al‐Bedah, 2013) and inflammatory factors such as C‐reactive protein (CRP) rise (Chistyakov et al., 2018). Vitamin B6, regardless of the level of serum, by acting on the blood levels of inflammatory factors and dopamine can reduce these factors and regulate serotonin levels (Spinneker et al., 2007; Ueland et al., 2017). By adjusting the above factors, the patient's symptoms of restlessness, aggression, mood swings, and psychosis may be better controlled with a prescription of vitamin B6. Due to the coenzyme role, increasing its amount, by increasing the level of PLP and increasing the activity of enzymes involved in the metabolism of various biochemical pathways, may lead to improved physiological and clinical functions (Zheng et al., 2018).

Given that bipolar disorder is a chronic disease and imposes a heavy economic burden on patients, families, and the community, and in addition, frequent recurrences provide the basis for more hospitalization of patients, any other alternative treatment that leads to improving symptoms can help reduce the burden of the disease. In this study, in addition to investigating the possible effect of vitamin B6 on bipolar disorder in manic episodes, we examined its effects on cognitive status, anthropometric changes, metabolic status and blood levels of inflammatory factors and homocysteine in a placebo‐controlled double‐blind clinical trial at Roozbeh Hospital in Tehran.

## 2 PATIENTS, MATERIALS, AND METHODS

### 2.1 Trial design and setting

This study is a randomized, 8‐week, double‐blind, placebo‐controlled, parallel‐group study of the adjuvant effect of vitamin B6 compared with placebo (in conjunction with standard lithium therapy) in the treatment of acute manic episode of type 1 bipolar disorder with psychotic feature. It was performed in Roozbeh Hospital in Tehran between May 2019 and June 2020.

### 2.2 Participants

Participants in the study included men and women between the ages of 18 and 65 who were diagnosed with type 1 bipolar disorder in the manic episode of the disease and with psychotic features according to the Diagnostic and Statistical Manual of Mental Disorders‐Fifth Edition (DSM‐5). They were considered for the study following a structured clinical interview. They were included in the study if they had a score of at least 20 based on the Young Mania Questionnaire, and after obtaining informed written consent from the patients and/or their caregiver.

Exclusion criteria included having active liver or heart disease and chronic kidney disease (CKD), history of neurological disorder and seizures, history of drug allergy to lithium carbonate or vitamin B6, presence of state delirium, anorexia nervosa, bulimia nervosa, history of any mental disorder other than bipolar disorder, history of recent alcohol or substance use (at least 3 months ago), IQ < 70, and pregnancy and lactation.

### 2.3 Intervention

Patients who entered the study based on the inclusion criteria and after rejecting the exclusion criteria and obtaining informed consent, were divided into one of two groups receiving vitamin B6 or placebo, based on randomization method and using a random number table. One group received 80 mg of vitamin B6 daily and the other group received placebo. The therapist and the patient were not aware of the type of the groups. Standard treatment of lithium did not stop in both groups. All patients received lithium (gradually increasing the dose to a therapeutic level of 0.8 to 1.2). No other treatments including Electroconvulsive therapy (ECT) or other treatments were performed on the patients. The clinical trial lasted for 8 weeks.

### 2.4 Study procedure

Before starting the treatment and as a baseline, all patients were evaluated for the severity of mania symptoms according to Young Mania criteria. Outcomes were measured in weeks 2, 4, and 8. In addition, in the mentioned weeks, patients were assessed in terms of cognitive status based on the criteria of the Mini‐Mental State Examination (MMSE) questionnaire. They were also assessed for appetite status using a simple appetite questionnaire during the mentioned weeks. Anthropometry with body composition analyzer and sleep status (using the Petersburg questionnaire) was also evaluated during the mentioned weeks as secondary outcomes. Another measure performed on patients was laboratory tests prior to enrollment with an assessment of fat profile status, inflammatory factors including erythrocyte sedimentation rate (ESR) and CRP, and measurement of blood levels of homocysteine, folic acid, and vitamin 12. All of these laboratory tests were re‐measured and evaluated at the end of 8 weeks.

### 2.5 Outcome

Patients were evaluated in terms of laboratory tests in weeks 0 and 8. Other parameters were evaluated in weeks 0, 2, 4, and 8. The primary purpose of this study was to evaluate the efficacy of vitamin B6 in improving the symptoms of bipolar disorder in manic episode in comparison with placebo based on the changes in the Young Mania Questionnaire score and related statistical analysis.

Comparative study of cognitive symptom improvement based on MMSE questionnaire, sleep state improvement based on Petersburg questionnaire, appetite status based on simple appetite questionnaire, anthropometric conditions, fat profile, and laboratory tests compared to control group based on statistical criteria were other objectives of this study. During the study, patients were evaluated for adverse drug events by a side‐effect checklist.

### 2.6 Sample size

Based on previous studies, due to the equality of variances between the two groups, a standard deviation of 3, a two‐sided significance of 5%, and a power of 80%, the required total sample size of 32 subjects was obtained. Assuming about 20% attrition rate and in order to ensure a sufficient sample size, finally 50 subjects (25 in each group) were considered as the final sample size.

### 2.7 Randomization, allocation concealment, and blinding

In order to randomize the participants, a computer random number generator was used so that the participants received one of the vitamins B6 or placebo in equal and one to one ratio in blocks of four. The drugs were concealed using numbered packages. Randomization and assignment were done by a person who had no involvement in the study. None of the participants in the study knew how the drugs were allocated. This included physicians, nurses, patients, statisticians, and investigators who rated the patients and prescribed drugs.

### 2.8 Ethics

The study was conducted in accordance with the provisions of the Helsinki Declaration. This study was performed based on the code of ethics obtained from Tehran University of Medical Sciences with the number IR.TUMS.MEDICINE.REC.1397.935 and the registration code number IRCT20200317046801N1 related to the registration of the study protocol of Iranian clinical trials. Written consent was obtained from all participants in the study and their caregivers to participate in the study. Participants could leave the study at any time during the study.

### 2.9 Scale

#### 2.9.1 Young Mania Questionnaire

This questionnaire, as one of the most widely used scales for measuring mania syndrome, has 11 items. The assessment is made through the patient's statements about his or her clinical condition over the past 48 h. Other information is also obtained through observations made during the clinical interview (Young et al., 1978). In evaluating the validity of the above questionnaire in Iran, Cronbach's alpha coefficient was 0.72, which indicates its acceptability for use in the Iranian population. Cutting points, sensitivity and specificity were 12.5, 0.93, and 0.96, respectively (Z. Mohammadi et al., 2018).

#### 2.9.2 Mini‐Mental State Examination

MMSE is a tool for quantifying the intensity of cognitive impairment and a tool for recording cognitive changes over time (Tombaugh & McIntyre, 1992). In evaluating the validity of the above questionnaire in Iran, good reliability and validity were obtained (Cronbach's alpha coefficient equal to 0.78) and at the cut‐off point of 21, showed a sensitivity of 90% and a specificity of 84% (Foroughan et al., 2008).

#### 2.9.3 The Pittsburgh Sleep Quality Index (PSQI)

PSQI has been developed by Buysse et al. (1989) as one of the best tools for measuring sleep quality. This questionnaire has 7 subscales and the internal consistency of the questionnaire based on Cronbach's alpha is 0.83 (Buysse et al., 1989). The Persian version of this questionnaire is acceptable for use in the Iranian population. In one of the related studies, the Cronbach's alpha coefficient for the control group was 0.78 and in the group of patients with psychiatric disorders was 0.52. Also, at cutting point 5, it showed 94% sensitivity and 72% specificity (Moghaddam et al., 2012).

#### 2.9.4 Simple appetite questionnaire

Wilson et al. (2005) used simple appetite questionnaire to assess loss of appetite among nursing home residents and community‐dwelling adults (Wilson et al., 2005). This questionnaire is directly related to the total calories in the diet. (*r* = .23, *p* = .018) It also shows a positive correlation with body weight, waist circumference and body fat percentage (convergent validity), and a negative correlation with muscle mass (divergent validity). Internal consistency of the test was confirmed by Cronbach's alpha coefficient of 0.7 (M. Mohammadi et al., 2019).

### 2.10 Statistical analysis

The SPSS 25 (IBM Corporation, Armonk New York) was used for data analysis. We compared the numbers and percentages with Chi‐square or Fisher Exact tests for categorical variables. Furthermore, we reported the mean ± standard deviation (SD) and used independent sample *t*‐test to compare the mean score changes of Young Mania and MMSE scores and lab test between the two groups from baseline to the trial endpoint. Kaplan‐Meier estimation with the log‐rank test was used to compare the time required to respond to treatment between the two groups.

The status of anthropometric changes and sleep quality index based on related scores were compared for the two groups in weeks 2, 4, and 8 using a mixed repeated‐measures analysis of variance (ANOVA) with Greenhouse–Geisser correction to account for non‐equal variances. Post hoc tests were performed, and for these tests, the Bonferroni correction was applied. Here, between‐subject factors were considered as two treatment groups and scores obtained in different weeks were considered as within‐subject factors.

In all data analysis, a two‐sided *p*‐value under .05 (*p*‐value < .05) was considered statistically significant.

## 3 RESULTS

**FIGURE 1:**
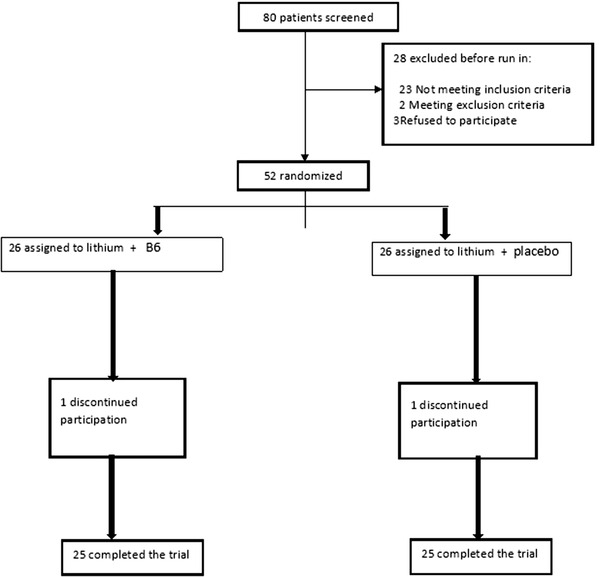
Flow diagram of the clinical trial of the efficacy of vitamin B6 as an adjunctive therapy in improving the symptoms of acute bipolar mania

The study flow diagram is shown in Figure 1. Demographic characteristics and baseline variables obtained in the study are listed in Table 1. As shown in this table, no significant differences were observed between the two groups in terms of demographic characteristics and baseline data. The scores obtained in both groups, based on the Young Mania Questionnaire and MMSE, are shown as Mean ± SD and statistical comparison by *t*‐test are shown in Table 2.

**TABLE 1:** Comparison of participants' baseline characteristics between two groups (patients with bipolar disorder, manic episode with psychotic feature, receiving vitamin B6 or placebo)

		Group 1 (vitamin B6) Group 2 (placebo) *p*‐value		
Age mean ± SD		30.96 ± 8.62	31.4 ± 11.88	.25
Male:Female		5:20	8:17	.33
Admit number history		2.92 ± 1.57	2.80 ± 1.70	.72
Electroconvulsive therapy history		12	7	.14
Self‐mutilation history		13	11	.57
Suicide attempt history		8	9	.55
Age of first episode		22.04 ± 5.82	21.08 ± 5.27	.4
Smoking		15	11	.25
Substance use history		3	8	.08
Alcohol use history		2	5	.26
Suicide number history		0.37 ± 0.64	0.52 ± 0.79	.64
Duration of disease (year)		8.56 ± 6.27	9.88 ± 9.82	.25
Education	Illiterate	0	1	.24
	Primary	10	12	
	High school	8	10	
	Higher education	7	2	
Marriage	Single	13	19	.23
	Married	7	5	
	Divorced	1	0	
	Widowed	4	1	
Occupation	Employed	0	1	.08
	Unemployed	1	6	
	Student	0	1	
	Other	24	17	
Internal medicine disease	DM	2	1	.11
	HTN	2	3	
	DM & HTN	2	2	
	Seizures	1	1	
	Hypothyroid	3	2	

**TABLE 2:** Comparison of Mania & MMSE score changes between two groups using independent *t*‐test

	Week	Group 1 (vitamin B6)	Group 2 (placebo)	CI95	*T*	*p*‐value
Young mania	0	50.60 ± 5.19	48.44 ± 6.36	−1.14–5.46	1.31	.19
	2	38.64 ± 6.97	36.44 ± 7.01	−1.77–6.17	1.11	.27
	4	28.36 ± 5.7	27.76 ± 4.89	−2.42–3.62	.39	.69
	8	22.68 ± 5.39	21.80 ± 5.39	−1.78–3.55	.66	.51
MMSE	0	17.95 3.09	18 ± 2.32	−1.61–.52	−.05	.27
	2	20.72 ± 3.83	20.56 ±2.46	−1.67–1.99	.17	.03*
	4	22.36 ± 3.35	23.40 ± 2.06	−2.62–.54	−1.32	.03*
	8	24.40 ± 3.25	25.24 ± 1.96	−2.36–.68	−1.1	.01*

*statistically significant.

Accordingly, in all study weeks, based on Yang Mania scoring scale, no significant difference was observed between the two groups. Based on MMSE in weeks 2, 4, and 8, significant improvement in cognitive status was obtained in group placebo compared to group vitamin B6 (The *p*‐value was .03, .03, and .01 in weeks 2,4, and 8, respectively). The status of the two groups of patients in terms of laboratory test results is shown as Mean ± SD and compared with *t*‐test in Table 3. In all lab tests, except for blood triglyceride level at week 8, no significant difference was observed between the two groups. The mean triglyceride level at the beginning of the study in the vitamin B6 group was 117.87 ± 49.14 and in the placebo group was 145.48 ± 68.74 and in week 8 the mean in the vitamin B6 group was 127.33 ± 59.83 and in the placebo group was 146.32 ± 61.47. Less increase in the placebo group compared to the vitamin B6 group was also statistically significant (*p*‐value = .007).

**TABLE 3:** Comparison of lab tests changes between two groups using independent *t*‐test

	Week	Group 1 (vitamin B6)	Group 2 (placebo)	*p*‐value
HB	0	13.20 ± 1.74	13.36 ± 1.15	.7
	8	13.48 ± 1.36	13.41 ± 1.10	
WBC	0	6308 ± 1557	6258 ± 1359	.95
	8	5992 ± 904	6064 ± 909	
FBS	0	84.95 ± 10.35	95.16 ± 14.14	.31
	8	91.84 ± 13.37	97.87 ± 15.88	
BUN	0	20.52 ± 5.45	97.87 ± 15.88	.24
	8	21.08 ± 3.76	21.56 ± 4.01	
TG	0	117.87 ± 49.14	145.48 ± 68.74	.007*
	8	127.33 ± 59.83	146.32 ± 61.47	
Total cholestrol	0	178.68 ± 47.39	190.04 ± 48.42	.92
	8	165.68 ± 56.81	177.72 ± 64.00	
HDL	0	43.20 ± 5.18	44.04 ± 4.38	.35
	8	41.25 ± 3.94	41.25 ± 3.94	
LDL	0	108.32 ± 34.9	110.32 ± 29.73	.63
	8	107.00 ± 33.30	111.68 ± 35.17	
AST	0	23.39 ± 7.06	24.16 ± 7.68	.99
	8	21.28 ± 5.91	21.96 ± 6.55	
ALT	0	21.91 ± 10.23	22.87 ± 10.68	.73
	8	19.44 ± 6.64	21.08 ± 6.40	
ALKP	0	177.60 ± 46.18	171.31 ± 49.78	.27
	8	180.80 ± 39.85	185.69 ± 55.18	
ESR	0	12.54 ± 11.44	9.98 ± 6.96	.67
	8	6.43 ± 3.96	4.68 ± 2.79	
Cr	0	0.94 ± 0.14	0.94 ± .11	.08
	8	0.97 ± 0.10	0.94 ± 0.13	
Serum level of folic acid	0	10.43 ± 2.13	12.51 ± 3.95	.96
	8	11.80 ± 3.03	12.51 ± 3.95	
Serum level of vitamin B12	0	505.84 ± 93.90	367.32 ± 83.44	.15
	8	500.32 ± 124.21	388.15 ± 71	
Homocysteine	0	13.45 ± 3.28	15.33 ± 4.86	.95
	8	12.32 ± 3.25	14.48 ± 4.50	
TSH	0	1.75 ± 1.03	2.31 ± 0.91	.12
	8	1.98 ± 1.06	2.48 ± 1.09	
T4	0	8.34 ± 1.38	7.53 ± 2.49	.83
	8	8.66 ± 1.26	7.32 ± 2.27	

*statistically significant.

Regarding anthropometric status, data analysis of this part of the study was performed using Repeated measure ANOVA (analysis of variance with multiple measurements). Table 4 shows the scores of the two groups of patients in terms of Mean ± SD that in all situations except muscle mass in the second week, no significant difference was observed between the two groups. At week 2, for muscle mass, the mean of the vitamin B6 group was 26.50 ± 4.26 and for the placebo group, the mean was 27.49 ± 5.36 with a *p*‐value of .001 compared to the two groups. There was no significant difference in this item in other weeks.

**TABLE 4:** Comparison of participants' characteristics between two groups using repeated measures

	Week	Group 1 (vitamin B6)	Group 2 (placebo)	*p*‐value
Muscle mass	0	26.54 ± 4.37	26.56 ± 4.63	—
	2	26.50 ± 4.26	27.49 ± 5.36	.01*
	4	26.55 ± 4.25	26.30 ± 3.96	.59
	8	26.59 ± 4.31	26.45 ± 3.99	.77
Fat%	0	33.14 ± 10.34	31.92 ± 10.08	—
	2	33.73 ± 10.83	31.84 ± 9.23	.36
	4	34.02 ± 10.97	31.68 ± 9.26	.16
	8	34.17 ± 10.97	31.58 ± 9.33	.15
Metabolism	0	1497 ± 235	1474 ± 179	—
	2	1490 ± 231	1497 ± 183	.14
	4	1482 ± 193	1507 ± 199	.18
	8	1482 ± 188	1507 ± 186	.31
Abdominal fat	0	5.88 ± 1.96	6.00 ± 2.48	—
	2	5.92 ± 1.91	5.96 ± 2.47	.6
	4	5.60 ± 2.16	6.08 ± 2.53	.33
	8	5.96 ± 1.92	6.04 ± 2.50	.56
Appetite	0	8.36 ± 4.19	9.92 ± 5.37	—
	2	10.28 ± 3.44	11.12 ± 4.75	.24
	4	11.08 ± 3.35	12.40 ± 4.50	.78
	8	12.20 ± 3.58	13.64 ± 4.30	.9
Weight	0	74.55 ± 16.23	70.40 ± 13.21	—
	2	75.47 ± 16.27	70.60 ± 16.42	.6
	4	76.56 ± 16.09	72.28 ± 13.81	.76
	8	77.59 ± 16.22	72.74 ± 13.93	.36
Height	0	163.48 ± 6.67	164.00 ± 6.53	.7
BMI	0	28.06 ± 5.98	26.15 ± 5.03	—
	2	28.03 ± 6.13	26.53 ± 5.09	.26
	4	28.52 ± 6.13	26.91 ± 5.04	.45
	8	29.05 ± 6.24	27.13 ± 5.11	.96

*statistically significant.

Also, in terms of appetite status, based on the simple appetite questionnaire, no significant difference was found between the two groups during the study. Sleep quality index was analyzed based on a statistical analysis of repeated measure ANOVA (analysis of variance with multiple measurements). The scores of the two groups of patients in terms of sleep quality using the Petersburg questionnaire as Mean ± SD are shown in Table 5. In the total score, in the second week, group vitamin B6 with a score of 1.92± 0.81 and group placebo with a score of 2.32 ± 5.19 with a *p*‐value equal to .04 had a statistically significant difference, but in the following weeks, no statistical difference was seen between the two groups. Regarding the mean score related to sleep quality in week 4, group vitamin B6 had a score of 1.36 ± 0.56 and group placebo had a score of 0.80 ± 0.57 with a *p*‐value of .03 and in week 8, the average of the group one was 1.08 ± 0.27 and group placebo was 0.44 ± 0.50 with *p*‐value equals to .02. Based on these results, sleep quality in group vitamin B6 was more appropriate than group placebo. Regarding the mean score related to sleep quality in week 4, group vitamin B6 had a score of 1.36 ± 0.56 and group placebo had a score of 0.80 ± 0.57 with a *p*‐value of .03 and in week 8, the average of the group vitamin B6 was 1.08 ± 0.27 and group placebo was 0.44 ± 0.50 with *p*‐value of .02. Also, sleep duration in weeks 2 and 4 in group placebo was better than group vitamin B6, which was also statistically significant (*p*‐value in both situations, equal to .03). Sleep disruption was statistically and significantly better in group placebo in the second week than in group vitamin B6 (*p*‐value equal to .01), but this difference was not seen in the following weeks between the two groups. No side effects due to medication/placebo were observed in the study.

**TABLE 5:** Comparison of participants' Pittsburgh Sleep Quality Index between two groups using repeated measures

	Week	Group 1 (vitamin B6)	Group 2 (placebo)	*p*‐value
Pittsburg (total)	0	2.36 ± .75	3 ± 4.84	—
	2	1.92 ± .81	2.32 ± 5.19	.04*
	4	1.40 ± .57	.8 ± .57	.12
	8	1.04 ± .20	.48 ± .50	.23
Pittsburg (quality)	0	2.28 ± .73	2.16 ± .68	—
	2	1.80 ± .86	1.48 ± .65	.29
	4	1.36 ± .56	.80 ± .57	.03*
	8	1.08 ± .27	.44 ± .50	.02*
Pittsburg (sleep latency)	0	2.04 ± .93	1.72 ± .67	—
	2	1.80 ± .81	1.24 ± .43	.18
	4	1.24 ± .72	.92 ± .49	1
	8	.88 ± .52	.64 ± .48	.75
Pittsburg (efficiency)	0	2.48 ± .65	1.72 ± .61	—
	2	1.84 ± .89	1.08 ± .49	1
	4	1.36 ± .48	.6 ± .5	1
	8	1.04 ± .2	.4 ± .5	.48
Pittsburg (sleep duration)	0	3.82 ± 1.02	3.80 ± .70	—
	2	4.50 ± 1.06	4.80 ± .91	.03*
	4	5.44 ± 1.19	5.84 ± 1.14	.03*
	8	6.36 ± 1.46	6.52 ± 1.47	.39
Pittsburg (sleep disturbances)	0	2 ± 1	1.52 ± .58	—
	2	1.64 ± .95	1.04 ± .53	.43
	4	1 ± .70	.60 ± .57	.64
	8	.60 ± .57	.32 ± .47	.30
Pittsburg (dysfunction)	0	1.68 ± 1.10	1.56 ± .65	—
	2	1.48 ± .96	.88 ± .60	.01*
	4	.84 ± .80	.56 ± .50	.42
	8	.48 ± .50	.24 ± .43	.59
Pittsburg (%) (medications to sleep)	0	100	100	—
	2	100	100	—
	4	88	100	.07
	8	76	68	.52

*statistically significant.

## 4 DISCUSSION

In this double‐blind, randomized, placebo‐controlled, clinical trial, the use of vitamin B6 along with lithium had no statistically significant effect on the improvement of bipolar mania symptoms. Also, there was no significant difference in terms of anthropometric status between the two groups. However, sleep quality was better among the vitamin B6 group than the placebo group.

For about 70 years since the use of lithium to treat mood disorders, attention to the use of minerals and vitamins as adjunctive therapy for these disorders has always been discussed (Popper, 2001). The use of high doses of vitamin diets has been one of these efforts, which has been associated with positive therapeutic results in some clinical conditions, such as reducing anxiety (Carroll et al., 2000). Assuming minor global micronutrient deficiencies, various studies have examined the effect of improving this condition on mood status among different groups of the general population. In this regard, for example, although some interventional studies have reported that B vitamins reduce clinical depression, other studies have not shown such an effect (Long & Benton, 2013). Cochrane study on the effect of vitamin B6 and folic acid in reducing the symptoms of clinical depression was not associated with positive results (Malouf & Evans, 2003). The first study was conducted by Bryan et al. (2002) In 2002.In their study, they compared 12 healthy women between the ages of 65 and 92 with 75 mg of vitamin B6 daily and compared them with 21 other healthy women with similar conditions in terms of cognitive status and mood after 5 weeks of medication/placebo. No clinical improvement in mood was reported, with only a slight improvement in functional memory in the vitamin B6 group versus placebo. The results were unexpected due to the initial hypotheses before the study, including the effect of vitamin B6 on the methylation process (Unnikrishnan et al., 2018). They attributed such a result to the lack of a dose–response relationship for vitamin B6 and mood and cognitive symptoms. They also stated that one of the reasons for such results was the possible short duration of the study and emphasized the need to continue such studies to reach a more complete conclusion.

In another study, Deijen et al. (1992) examined 76 healthy men between the ages of 70 and 79. They randomly administered 20 mg of vitamin B6 to a group of 38 people daily for 12 weeks and gave a placebo to the other group (Deijen et al., 1992. In the end, the two groups did not have a significant difference in terms of mood. Attempting to achieve appropriate doses of vitamin B6 with a possible effect on mood and cognition was one of the points of interest of researchers in this study.

The results of our study are close to the results of these two studies. In our study, 50 patients with bipolar disorder in the acute phase of mania with psychotic feature were randomly assigned to receive 80 mg of vitamin B6 daily or a placebo. The final results of our study after 8 weeks of daily drug treatment showed no change in mood in patients treated with vitamin B6 compared with patients treated with placebo. Also, in our study, the group receiving the drug, in terms of cognitive status, did not have a more appropriate status than the other group. Statistical significance can be considered as a placebo effect (Kaptchuk & Miller, 2015) or a random finding (Wolf et al., 2008).

The treatment for bipolar disorder is based on mood stabilizers (Sadocket al., 2017). Lithium is widely used as the main drug to control this disorder in the acute and chronic period of this disorder (Jope, 1999). Despite the definite usefulness of lithium therapy in this disorder, this drug accompany with the important complication of weight gain and increase in body mass index and changes in leptin levels (Atmaca et al., 2002), abnormal state of metabolic status and impairment of fat profile (Livingstone & Rampes, 2006) and some degree of cognitive impairment (memory impairment) (Pachet & Wisniewski, 2003).

In addition, despite the evidence in favor of the anti‐inflammatory effects of lithium, some studies have reported inflammatory conditions in the use of this drug in some conditions (Nassar & Azab, 2014). Regarding the metabolic effects of this drug, this situation is also very obvious in our study and patients with any metabolic and anthropometric conditions, from the beginning of treatment with lithium had an increase in these indicators. In our study, in both groups, we face an increase in mean triglyceride as one of the main criteria of metabolic syndrome (Després & Lemieux, 2006).

Also in our study at the end of 8 weeks in both groups we see a decrease in mean HDL, which is one of the main indicators of metabolic syndrome. Also, abdominal fat, appetite, weight and body mass index in both groups increased with increasing duration of lithium use, regardless of having or not having a previous history of using this medication. The findings of many meta‐analyzes of the status of metabolic syndrome and other metabolic disorders in bipolar patients strongly support the claim that patients with bipolar disorder are at risk for metabolic syndrome, cardiovascular complications, and mortality. They recommend the need for regular monitoring and preventive measures and appropriate treatment for cardiac and metabolic risk factors (Vancampfort et al., 2013).

As mentioned in the previous sections, bipolar disorder, especially in recurrent conditions such as the mania phase, causes inflammation (Rosenblat & McIntyre, 2016), increased levels of inflammatory factors (Dargél et al., 2015), and elevated levels of homocysteine (Salagre et al., 2017) as a pro‐inflammatory factor.

In our study, ESR levels were significantly reduced in both groups at the beginning and end of 8 weeks of treatment, so that at the end of 8 weeks, this level reached nearly half of the baseline level in both groups. This is also true for the mean serum level of homocysteine, and after 8 weeks, a decrease in serum levels was observed in both groups.

However, in our study, the reduction in serum homocysteine levels after receiving 8 weeks of treatment with 80 mg of vitamin B6 was not statistically significant compared to the placebo group and the difference between the two groups was not statistically significant. Also, there was no change in the inflammatory status of patients between the two groups, and finally, there was no change in the severity of mania symptoms in the group receiving the medication, compared to the placebo group.

Vitamin B6 is involved in the production cycle of serotonin and melatonin from tryptophan and is thus one of the most important micronutrients involved in the sleep and mood of patients (Rodwell et al., 2015). However, in our study, the group that received daily vitamin B6 did not generally sleep better than the placebo group.

However, according to the Petersburg questionnaire, in the sleep quality subscale, the vitamin B6 group experienced a better situation than the placebo group at the end of 8 weeks of study. These conditions can emphasize the important point that the probable clinical effects due to vitamin B6 may not commensurate with the serum level, and various other factors may be involved.

In addition to the above, one of the points that can be raised in this regard is the clinical condition of the patients under study. These patients have a higher severity of the disease than other patients who do not have psychotic manifestations due to their psychotic feature along with mood manifestations. In this group of people, the response to treatment is usually later and the duration of acute symptoms is longer, and therefore sleep improvement may be more delayed (Özyıldırım et al., 2010).

Also, the effects of supplements such as vitamin B6 may appear late than normally expected or even in conditions different from what is expected from serum levels.

In a study of 12 healthy men by Luboshitzky et al. (2002) at 5 p.m., half of them were given 100 mg of vitamin B6 and the other half were given placebo. At about 10 p.m. in both groups receiving the drug and placebo, the serum level of melatonin increased without any statistical difference between the two groups. Also, the amount of sleep pattern between the two groups was the same (Luboshitzky et al., 2002). In their description, they noted the increase in serotonin and melatonin levels in children's age groups, as well as in some animal studies, following intravenous administration of vitamin B6. They cited the possible reason for the inconsistency of the results of their study with similar studies, the difference in age of the subjects, the dose of pyridoxine used, and the different design of the two studies.

They also cited oral administration of vitamin B6 as a possible link to delayed drug uptake as well as delayed hepatic metabolism and attributed this to the lack of access to a more effective serum level of the vitamin.

They also cited the health of the study group as another reason for not responding to the prescribed vitamin B6 (especially because of their previous adequate levels of the drug), noting that they may not have used higher levels of tryptophan and pyridoxine.

In another study, Aspy et al. (2018) in Australia examined sleep patterns among 100 people who were given 240 mg of vitamin B6 daily for 5 consecutive days and reported only an increase in the recall of dream components. They did not report any change in the quality or other sleep‐related variables following receiving this dose (Aspy et al., 2018). As mentioned, in our study, patients in the group treated with vitamin B6 reported better sleep quality than patients receiving placebo, which was also statistically significant. However, there was no statistically significant difference between the two groups in other components related to sleep.

Thus, there seems to be a lot of complexity in interpreting the effect of variable doses of vitamin B6 and its duration of use and serum levels, on the quality and other components of sleep, among healthy individuals and psychiatric patients. It seems that conducting different studies in different conditions in terms of dose and duration of use and multicenter studies and interpretation of the results in the form of systematic reviews can help to understand the effects of vitamin B6 on the sleep status of patients with bipolar disorder.

In a study conducted by Nauruth et al., the effect of vitamin supplements in the form of transmission by intramuscular injection of 8 injections over 3 weeks at a dose of 1 mg of vitamin B12, 1.1 mg of folate, and 5 mg of vitamin B6 for two groups of elderly patients living at home (*n* = 175) and hospitalized patients (*n* = 110) were evaluated (Naurath et al., 1995). They also measured serum levels of homocysteine, methyl malonic acid, 2‐methyl citric acid, and cystathionine during the 3 weeks of the study, and reported a decrease in all four metabolites at the end of the study, especially the highest relative decrease on days 5 to 12 in the supplement group compared with the placebo–control group.

The interesting point in this report was the decrease in homocysteine level compared to the beginning of the study in both groups, which was higher in the supplement group (92% decrease compared to 20% decrease in the supplement and placebo groups, respectively, compared to the beginning of the study). Referring to the rate of response to vitamin supplements (in this study in the elderly), in reducing harmful metabolites, they stated that metabolic evidence of vitamin deficiency is common in the elderly, even in the presence of natural serum vitamins, and this can be an indication of the need to take vitamin supplements including vitamin B6 in some groups, even with normal levels of vitamins.

In our study, homocysteine levels were measured before and after drug/placebo intervention in all patients. In group one (receiving medication), this rate was equal to 8% reduction in serum levels at the end of the study compared to the beginning of the study, which in group two (receiving placebo) was equal to 6% reduction in serum levels.

In this section, we can point out a few points. The first point is the placebo effect in both studies. In the study of Nauruth et al., this effect was equal to 20% and in our study, it was equal to 6%. Another important point is the comparative study of the decrease in homocysteine levels in our study and the mentioned study (8% vs. 92%, respectively). One of the important differences between this study and our study is that despite the use of lower doses of vitamin B6 and less study time (3 weeks vs. 8 weeks in our study), the rate of decrease in homocysteine blood levels has been obviously achieved in a higher level.

One of the points that may explain such a difference is the coexistence of other vitamin supplements, including vitamin B12 and folic acid in that study. In our study, in order to ensure that the conditions of the two groups were the same in terms of vitamin B12 and folic acid levels, their serum levels were evaluated before and after the study, but no significant difference was found between the two groups. This was particularly important given the effect of vitamin B12 and folic acid deficiency on increasing homocysteine levels (Brattström et al., 1988; Zhang et al., 2009).In our study, the use of relatively high daily doses of vitamin B6 was not statistically associated with a final reduction in homocysteine levels compared to the other group of bipolar patients. This can be one of the reasons for the lack of final improvement in the Yang Mania questionnaire scores in both groups. Another point that can be seen in the study is that the average result of homocysteine tests in the first group is at the level closest to the normal maximum and in the second group is above the normal level. This finding is consistent with similar findings in other studies (Ozdogan et al., 2020).

Other points that can be addressed in the use of vitamin B6 in the studied patients are the lack of improvement in body mass index status and other anthropometric criteria, as well as appetite status between the two groups in which the difference between the two groups was statistically insignificant. All the mentioned components in both groups at the end of the study (anthropometric measurements, and metabolic status) were in a more unfavorable condition than at the beginning of the study. There was no statistically significant difference between the two groups.

Also, in the control group compared to the group receiving vitamin B6, the rate of increase in serum triglyceride levels was lower, which was statistically significant.

One of the primary goals of this study was to use vitamin B6 as an adjunct therapy to control the patient's mood and at the same time to regulate the anthropometric status of patients. In our study, despite initial evidence, vitamin B6 had no effect on the improvement of these components at the end of 8 weeks. In this regard, in analyzing such results, we can consider the possibility of short study duration, sample size used and age and gender status of patients before the study.

In a study of low‐income men over the age of 50 in Beirut, 85 patients with high serum triglyceride levels (greater than 150 mg/L) were randomly divided into five groups. They were treated daily with various supplements for 12 weeks. Five groups were treated with lysine (1 g daily), vitamin B6 (50 mg daily), a combination of these two, carnitine (1 g daily), and placebo. They reported a 10% decrease in cholesterol and a decrease in triglyceride to 36.6 mg/dl at week 6 only among those receiving vitamin B6 alone, and an increase in triglyceride to 18 mg/dl in the placebo group. They did not describe it as statistically significant (Hlais et al., 2012).

As mentioned, in our study, in terms of anthropometric indices, there was no statistically significant difference between the two groups.

Also in our study, the amount of total cholesterol in the group receiving vitamin B6 decreased by 8% and in the group receiving placebo by 7% at the end of 8 weeks. However, there was no statistically significant difference between the two groups. Regarding blood triglyceride levels, this amount increased by about 10 mg/ dl in the vitamin B6 group and about 1 mg/dl in the placebo group, which was also statistically significant (*p*‐value = .007). Thus, a lower increase in triglyceride was obtained in the placebo group than in the group treated with vitamin B6. Further reduction of cholesterol was obtained in the group treated with vitamin B6 compared to the placebo group, but it was not statistically significant.

Due to the large differences in the results of studies, it seems necessary to continue studies in this field by conducting multicenter studies, with a larger sample size and taking into account factors such as age, sex and anthropometric conditions, and other factors involved in this process. As can be seen in the studies, the groups under study do not have sexual uniformity, age, underlying disease, and associated drugs.

## 5 CONCLUSION

According to this study, the daily dose of 80 mg of vitamin B6 for 8 weeks in patients with bipolar disorder in the manic episode with psychotic feature treated daily with lithium, was not associated with a significant improvement in mood status compared to the control group. Regarding fat profile, the increase in blood triglyceride occurred less in the placebo group than in the vitamin B6 group, although in both groups we saw an increase in triglyceride at the end of the study compared to the beginning of the study. Total cholesterol levels in both groups decreased at the end of the study, but there was no significant difference between the two groups.

Despite significant improvement in the level of inflammatory factors before and after the study, at the end of the study, there was no significant difference between the two groups. Homocysteine levels at the end of the study decreased in both groups, but there was no significant difference between the two groups. Body mass index, appetite and abdominal fat in both groups at the end of the study had increased, compared to before the study and at the end of the study, there was no statistically significant difference between the two groups.

Finally, according to our study, it seems that due to the high importance and the need to try to better control of the mood, cognitive status and anthropometric conditions of bipolar patients treated with lithium, especially in patients with psychotic features, studies with more sample size and longer duration are needed. Furthermore, combining vitamin B6 with other cofactors in the pathway of neurotransmitters synthesis could be suggested for future studies.

### 5.1 Limitations

Due to the selection of patients with psychotic feature in the study, collecting information about patients, especially in the first weeks of the study, was relatively difficult. These conditions were managed by establishing an appropriate rapport with patients and the cooperation of psychiatric nurses.

## FUNDING INFORMATION

Tehran University of Medical Sciences

## CONFLICT OF INTEREST

There is no conflict of interest.

### PEER REVIEW

The peer review history for this article is available at https://publons.com/publon/10.1002/brb3.2394

## Data Availability

The data that support the findings of this study are available on request from the corresponding author.
